# Assessing Auditory and Cochlear Function in Alopecia Areata Patients: Exploring the Link to Cochlear Melanocyte Damage

**DOI:** 10.7759/cureus.44882

**Published:** 2023-09-08

**Authors:** Aynur Aliyeva, Ozlem Yagiz Agayarov, Esra Inan Dogan

**Affiliations:** 1 Department of Otolaryngology-Head and Neck Surgery, Cincinnati Children's Hospital Medical Center, Cincinnati, USA; 2 Department of Otolaryngology-Head and Neck Surgery, Tepecik Training and Research Hospital, Health Sciences University, Izmir, TUR; 3 Department of Dermatology and Venereology, Adiyaman University Faculty of Medicine, Adiyaman, TUR

**Keywords:** auditory dysfunction, otoacoustic emission, cochlear melanocytes, melanasitis, alopecia areata

## Abstract

Introduction

Alopecia areata (AA) is an autoimmune disorder causing hair loss, including eyebrows, eyelashes, and body hair, primarily due to melanocyte impact. Though the precise AA melanocyte hearing loss mechanisms are not fully clear, it's speculated that cochlear melanocyte inflammation could disrupt endolymph production, which is necessary for sound signal transmission. Cochlear melanocytes maintain crucial potassium ion levels, which are pivotal for hearing. The potential AA-melanocyte-hearing loss link underscores the need to monitor auditory and cochlear function and consider interventions for AA-related hearing challenges. The study aimed to assess auditory and cochlear function using OAE and audiometry measurements to correlate disease severity and duration with OAE outcomes.

Materials and methods

In this study, we included 32 patients diagnosed with AA; the control group consisted of 29 healthy volunteers. We collected data on the patient's age, gender, onset age, family history, and disease duration. Audiological and otological evaluations were conducted, including pure tone audiometry (PTA), speech discrimination test (SD), and otoacoustic emission (DPOAE) measurements at frequencies of 500, 1000, 2000, 4000, 6000, 8000, and 10000 Hz. The patients were divided into two groups based on age: 18-25 and over 25 years old, and all parameters were compared. To examine differences between the right and left ears, gender, and age groups, we initially tested the variables for normal distribution using the Kolmogorov-Smirnov and Shapiro-Wilk tests. An independent sample t-test was conducted to compare the means for normally distributed variables.

Results

There were statistical differences at the 5% significance level in the mean DPOAE values of the 1 KHz SNR and 6 KHz SNR variables. According to the Mann-Whitney U test results, a significant difference was found in the gender-based DPOAE value at 2 kHz SNR (p=0.041), which was lower in men than women. Although there were no significant differences in the audiological parameters based on age, significant differences were found in the otoacoustic emission values. Variables, including 4 kHz DP1 (p=0.049), 500 Hz SNR (p=0.045), and 1 kHz SNR (p=0.023), differed significantly between age groups, with these values being lower in patients over 25 years old.

Conclusion

Overall, our study contributes to the growing body of evidence supporting an association between AA and auditory dysfunction, emphasizing the need for comprehensive assessment and management of hearing-related issues in individuals with AA.

## Introduction

Alopecia areata (AA) is an autoimmune disorder characterized by hair loss primarily affecting hair follicles. Various studies have reported a prevalence of 12% for coexisting autoimmune diseases with AA, including thyroid diseases, psoriasis, pernicious anemia, and vitiligo. Compelling evidence suggests that autoimmune targeting of follicular melanocytes plays a significant role in the development of AA [1-2].

The mechanisms underlying melanocyte damage in the skin can also impact other organs, such as the inner ear. For example, vitiligo, a condition that affects all active melanocytes, has been associated with auditory problems. Studies indicate that in vitiligo patients, hyperacusis ranges from 4% to 37%. The loss of epidermal melanocytes is believed to contribute to the development of vitiligo, and a similar loss of melanocytes in the inner ear can lead to sensorineural hearing impairment, compromising their essential functions and increasing susceptibility to environmental factors [2-4].

Interestingly, evidence suggests a potential link between AA and the inflammation of melanocytes in the cochlea. This inflammation is proposed as a mechanism connecting AA to hearing loss. The exact mechanisms underlying how melanocytes in AA contribute to hearing loss are not fully understood. However, it is speculated that inflammation and damage to melanocytes in the cochlea could disrupt their essential role in producing endolymph, the fluid necessary for transmitting sound signals to the auditory nerve. Melanocytes in the cochlea maintain high concentrations of potassium ions (K+) in the endolymph, which are vital for proper hearing function [4].

Melanocytes play a crucial role in the transduction and modulation of auditory signals in the inner ear. Previous research has suggested that melanocytes may serve practical and structural functions within the auditory system, which can be impaired in vitiligo. Recent studies have explored the potential association between AA and sensorineural hearing loss, proposing a hypothesis of autoimmune-mediated loss of follicular melanocytes [4-6].

It is plausible that AA affects hearing function by influencing melanocytes in the inner ear through a comparable mechanism. Therefore, this study aims to establish a link between AA and sensorineural hearing loss, further investigating the relationship between these conditions [6,7].

## Materials and methods

Our research was conducted as a prospective case-control study following the approval of the Adiyaman University Faculty of Medicine Ethics Committee N 2020/8-5 on 22.09.2020.

For this investigation, a cohort of 32 patients diagnosed with alopecia areata (AA) was enrolled alongside a control group comprising 29 individuals of sound health. The study encompassed comprehensive data collection pertaining to the patient's age, gender, age of onset, familial medical history, and duration of the ailment. Thorough audiological and otological assessments were executed, as were pure tone audiometry (PTA), speech discrimination tests (SD), and otoacoustic emission (DPOAE) measurements across a spectrum of frequencies: 500, 1000, 2000, 4000, 6000, 8000, and 10 000 Hz (DP1 and SNR). Stratification of patients was performed based on age, dividing them into two categories: 18-25 years old and above 25 years old, with subsequent comparative analysis of all parameters.

To ascertain distinctions between the right and left auditory channels, gender influences, and age divisions, a preliminary assessment of variable normality was executed via the Kolmogorov-Smirnov and Shapiro-Wilk tests. An independent sample t-test was performed for normally distributed variables to assess and compare means.

Exclusion criteria were rigorously applied, excluding individuals with histories of otologic diseases, familial susceptibility to hearing loss, use of systemic ototoxic drugs, presence of chronic systemic disorders, prolonged exposure to intense noise, a history of cranial trauma, prior diagnosis of autoimmune conditions, type B tympanogram (indicative of irregular middle ear function), air-bone conduction threshold discrepancies exceeding 5 dB, interaural threshold divergence of ≥15 dB, external auditory canal disorders, and other otological diseases.

Historical information regarding type 1 diabetes was collected. Also, essential blood tests were administered to gauge serum vitamin B12 levels, thyroid-stimulating hormone (TSH) levels, and concentrations of anti-thyroglobulin and anti-thyroid peroxidase antibodies. Only participants within the defined normal range for these parameters were included in the study.

Dermatological evaluation

A dermatology specialist at the same hospital conducted a comprehensive dermatological assessment of all patients. This encompassed an examination of various factors, including age, gender, disease duration, pattern and extent of alopecia (scalp hair, facial hair, eyebrows, generalized alopecia, etc.), classification of alopecia type (alopecia totalis, alopecia universalis, alopecia areata), severity of alopecia, and frequency of disease episodes. Furthermore, concomitant dermatological conditions like atopy and vitiligo were assessed.

Otologic and audiometric evaluation

A thorough otomicroscopic and tympanometric assessment was carried out by an otolaryngology specialist on participants from both the AA and the control groups. This meticulous evaluation aimed to identify any middle ear pathologies, with individuals displaying such conditions being subsequently excluded from the study.

The audiological evaluation encompassed pure tone audiometry and distortion product otoacoustic emissions (DPOAE) measurements, all administered by an expert audiologist within an acoustically controlled environment. Pure tone audiometry involved the determination of air conduction thresholds spanning frequencies from 250 Hz to 8,000 Hz for each ear, alongside bone conduction threshold levels ranging from 500 Hz to 4,000 Hz. DPOAE measurements were executed at 0.5 kHz, 1 kHz, 2 kHz, 4 kHz, 6 kHz, 8 kHz, and 10 kHz for both ears. The resulting data were scrutinized alongside signal-to-noise ratio (SNR) values, thereby facilitating a comprehensive evaluation of otoacoustic emissions.

## Results

No significant differences between the AA and control groups were observed in the audiometry parameters (p > 0.05) between the healthy volunteers and AA patients at 1,000 Hz, 2,000 Hz, 4,000 Hz, and 8,000 Hz frequencies. However, there were statistically significant differences at the 5% significance level in the mean DPOAE values of the 1 KHz SNR and 6 KHz SNR variables (p=0.03 and p=0.027). According to the Mann-Whitney U test results, a significant difference was found in the gender-based DPOAE value at 2 kHz SNR (p=0.041), with this value being lower in men than women (Tables 1, 2).

**Table 1:** Audiological Data Analysis by Gender (Group Statistics) Among the given variables, only the mean values of the 1KHzSNR and 6KHzSNR variables exhibit statistically significant differences at the 5% significance level.

Frequency (Hz)	Gender	N	Mean	Std. Deviation	Std. Error Mean
500HzDP1	Female	6	7.75	7.008	2.023
	Male	26	6.31	9.879	1.370
1KHzDP1	Female	6	11.17	9.514	2.746
	Male	26	6.37	7.968	1.105
2kHDP1	Female	6	8.83	5.289	1.527
	Male	26	5.94	8.912	1.236
4KHzDP1	Female	6	-2.83	7.941	2.292
	Male	26	-3.58	7.220	1.001
6KHzDP1	Female	6	-0.08	9.605	2.773
	Male	26	-5.29	8.601	1.193
8KHzDP1	Female	6	-6.33	5.852	1.689
	Male	26	-6.88	6.986	0.969
1KHzSNR	Female	6	16.08	8.806	2.542
	Male	26	9.00	6.857	0.951
6KHzSNR	Female	6	13.58	11.389	3.288
	Male	26	6.85	8.788	1.219
8KHzSNR	Female	6	6.08	5.600	1.616
	Male	26	5.42	6.557	0.909

**Table 2:** Independent Samples Test for the Audiological Data Analysis by Gender Among the given variables, only the mean values of the 1KHzSNR and 6KHzSNR variables exhibit statistically significant differences at the 5% significance level.

ncy (Hz)	t	df	Sig. (2-tailed)	Mean Difference	Std. Error Difference	95% Confidence Interval of the Difference
500HzDP1	Equal variance assumed	0.477	62	0.635	1.442	3.021
1KHzDP1	Equal variance assumed	1.814	62	0.074	4.801	2.646
2kHDP1	Equal variance assumed	1.077	62	0.286	2.891	2.685
4KHzDP1	Equal variance assumed	0.316	62	0.753	0.744	2.355
6KHzDP1	Equal variance assumed	1.850	62	0.069	5.205	2.814
8KHzDP1	Equal variance assumed	0.253	62	0.801	0.551	2.177
1KHzSNR	Equal variances assumed	3.054	62	0.003	7.083	2.319
6KHzSNR	Equal variances assumed	2.261	62	0.027	6.737	2.979
8KHzSNR	Equal variance assumed	0.322	62	0.748	0.660	2.049

Regarding the measurements of DPOAE, the analysis showed that there were no statistically significant differences detected at frequencies other than those specified between the individuals affected by AA and those belonging to the control group (p > 0.05). This comprehensive statistical examination encompassed a range of factors, including the localization of the disease (such as hair, beard, eyebrows, or the entire body), the type of alopecia, the severity of the condition, the frequency of its occurrences, and the presence of nail involvement.

The results of this in-depth analysis indicated that there were no noticeable distinctions observed in terms of inner ear involvement between the groups (p > 0.05). This suggests that the various parameters studied, such as the specific manifestations and characteristics of alopecia, did not appear to significantly impact inner ear function as assessed by DPOAE measurements. Therefore, this study suggests that while there may be associations between AA and auditory abnormalities in some cases, inner ear involvement, as indicated by DPOAE measurements, did not seem to be strongly influenced by the diverse aspects of the alopecia condition analyzed.

Although there were no significant differences in the audiological parameters based on age, significant differences were found in the otoacoustic emission values. Variables, including 4 kHz DP1 (p=0.049) and 1 kHz SNR (p=0.023), differed significantly between age groups, with these values being lower in patients over 25 years old by the independent test. Also, according to the Mann-Whitney U test results, the variable 500Hz SNR demonstrates a significant difference across age groups at a 5% significance level, which is also lower in patients over 25 years old (Tables 3-5).

**Table 3:** Audiological Data by Age Groups

Frequency (Hz)	Age Groups	N	Mean	Std. Deviation	Std. Error Mean
500HzDP1	18-25 Age	15	7.17	10.059	1.836
	25+ Age	17	6.06	8.849	1.518
1KHzDP1	18-25 Age	15	9.07	7.896	1.442
	25+ Age	17	5.68	8.647	1.483
2kHDP1	18-25 Age	15	8.07	8.191	1.496
	25+ Age	17	5.09	8.444	1.448
4KHzDP1	18-25 Age	15	-1.53	8.345	1.524
	25+ Age	17	-5.12	5.861	1.005
6KHzDP1	18-25 Age	15	-3.27	9.759	1.782
	25+ Age	17	-5.24	8.217	1.409
8KHzDP1	18-25 Age	15	-5.47	7.678	1.402
	25+ Age	17	-7.94	5.673	0.973
1KHzSNR	18-25 Age	15	12.63	7.122	1.300
	25+ Age	17	8.29	7.725	1.325
6KHzSNR	18-25 Age	15	8.73	11.004	2.009
	25+ Age	17	7.56	8.302	1.424
8KHzSNR	18-25 Age	15	6.37	6.718	1.227
	25+ Age	17	4.82	6.018	1.032

**Table 4:** Independent Samples Test for the Audiological Data by Age Groups Among the given variables, the 4KHzDP1 and 1KHzSNR variables exhibit statistically significant differences across age groups

Frequency (Hz)	t	df	Sig. (2-tailed)	Mean Difference	95% Confidence Interval of the Difference
500HzDP1	0.469	62	0.641	1.108	-3.616 to 5.832
1KHzDP1	1.630	62	0.108	3.390	-0.768 to 7.548
2kHDP1	1.428	62	0.158	2.978	-1.191 to 7.148
4KHzDP1	2.007	62	0.049	3.584	0.014 to 7.155
6KHzDP1	0.876	62	0.384	1.969	-2.523 to 6.461
8KHzDP1	1.478	62	0.145	2.475	-0.873 to 5.822
1KHzSNR	2.326	62	0.023	4.339	0.609 to 8.069
6KHzSNR	0.485	62	0.629	1.175	-3.663 to 6.012
8KHzSNR	0.969	62	0.336	1.543	-1.639 to 4.725

**Table 5:** Mann-Whitney U Test Statistics for the Audiological Data by Age Groups Grouping Variable: Age Groups. The table displays Mann-Whitney U test statistics for various variables.

Variable	Mann-Whitney U	Wilcoxon W	Z	Asymp. Sig. (2-tailed)
10KHz DPI	453.000	1048.000	-0.769	0.442
500HzSNR	361.000	956.000	-2.009	0.045
2kHSNR	381.500	976.500	-1.733	0.083
4KHzSNR	413.500	1008.500	-1.301	0.193
10KHzSNR	502.000	967.000	-0.108	0.914

## Discussion

Alopecia areata is a relatively common autoimmune disorder characterized by sudden hair loss. It affects individuals of all ages and genders, with a global prevalence estimated at around 2%. Various studies have reported a prevalence of 12% for coexisting autoimmune diseases with AA, including thyroid diseases, psoriasis, pernicious anemia, and vitiligo [1,2].

The exact cause of AA remains multifaceted, involving a complex interplay of genetic predisposition, immune dysregulation, and environmental triggers. It often presents within families with a history of autoimmune diseases, suggesting a genetic component. The central pathogenic mechanism in AA is the immune system's attack on hair follicles, leading to hair loss. Immune cells, particularly CD8+ T lymphocytes, infiltrate hair follicles, resulting in their miniaturization and eventual loss. AA is considered an organ-specific autoimmune disorder where the immune response targets hair follicles specifically [2,4,8,9]. The loss of immune tolerance and the subsequent attack on hair follicle antigens contribute to the condition's onset. The immune-mediated attack on hair follicles disrupts their normal growth cycle, causing them to enter a resting phase prematurely. This results in the abrupt shedding of hair and the formation of characteristic bald patches. Genetic factors play a substantial role in AA susceptibility. Genome-wide association studies have identified specific genetic variants associated with increased risk, linking the disorder to immune response genes [10].

Emerging research has explored potential links between AA and inner ear disorders, particularly hearing loss. Some individuals with alopecia areata report auditory issues, suggesting shared immune mechanisms impacting both systems. Although the relationship between AA and hearing loss requires further investigation, a plausible link exists due to the immune-mediated nature of both conditions. Autoimmune responses targeting hair follicles could potentially extend to inner ear structures [11]. The presence of autoantibodies against hair follicle antigens in AA patients suggests potential immune cross-reactivity. Shared antigens between hair follicles and inner ear components could lead to autoimmune damage in both systems [12]. Some individuals with alopecia areata may exhibit symptoms similar to autoimmune inner ear disease (AIED). AIED involves immune-mediated damage to inner ear sensory structures, implying common underlying autoimmune mechanisms [13].

Clinicians managing AA cases should be vigilant about monitoring hearing function. Auditory assessments can aid in the early detection of potential hearing loss, ensuring timely intervention if necessary.

Numerous investigations have highlighted a correlation between AA and sensorineural hearing loss (SNHL), particularly emphasizing higher frequencies. Regrettably, the significance of high-frequency SNHL often goes unnoticed, potentially presenting solely as tinnitus initially [11]. In a study conducted by Ucak and colleagues, a larger proportion of patients (54.9%) exhibited sensorineural disorders, surpassing the prevalence found in our study (23.8%) [14]. Another study demonstrated a significant correlation between cochlear dysfunction and the severity of alopecia areata based on the otoacoustic emissions test [15]. Contrarily, in our study, we observed no correlation within the audiometric parameters.

There are fewer number of manuscript that show the gender difference in hearing loss during AA. Research indicates that there might be differences in the pattern of hearing loss between genders in AA patients. For instance, males might exhibit more frequent involvement in specific frequency ranges or more pronounced sensorineural deficits than females [8,16]. Gender differences in hormonal levels, especially androgens, could potentially influence the auditory system's response to autoimmune processes in AA. Hormonal differences between males and females might contribute to variations in the prevalence or characteristics of hearing loss [17]. Our study finds statistically significant differences at the 5% significance level in the mean DPOAE values of the 1 KHz SNR and 6 KHz SNR variables (p=0.027 and p=0.03). According to the Mann-Whitney U test results, a significant difference was found in the gender-based DPOAE value at 2 kHz SNR (p=0.041), with this value being lower in men than women. Which can be related to hormonal factors. Our study highlights the potential connection between AA and hearing loss. Our findings indicate that there were statistically significant differences in certain otoacoustic emission values, and these findings support the notion that AA may impact cochlear function. Moreover, we observed a significant difference in gender based OAE values, with men exhibiting lower values compared to women. This suggests that gender may play a role in the potential auditory effects of AA. These results highlight the importance of monitoring auditory and cochlear function in individuals with AA, especially considering the potential risk of hearing difficulties.

## Conclusions

The presence of cochlear dysfunction in individuals with AA suggests that the presumed autoimmunity targeting follicular melanocytes in AA may also affect the melanocytes in the inner ear. This observation strengthens the relationship between sensorineural hearing loss and autoimmunity. Furthermore, this study in the literature represents the first documented evidence of hearing loss in AA using DPOAE.
